# Experimental Evolution Reveals Unifying Systems-Level Adaptations but Diversity in Driving Genotypes

**DOI:** 10.1128/msystems.00165-22

**Published:** 2022-10-13

**Authors:** Erol S. Kavvas, Christopher P. Long, Anand Sastry, Saugat Poudel, Maciek R. Antoniewicz, Yang Ding, Elsayed T. Mohamed, Richard Szubin, Jonathan M. Monk, Adam M. Feist, Bernhard O. Palsson

**Affiliations:** a Department of Bioengineering, University of California, San Diego, La Jolla, California, USA; b Department of Chemical and Biomolecular Engineering, University of Delaware, Newark, Delaware, USA; c Department of Pediatrics, University of California, San Diego, La Jolla, California, USA; d Novo Nordisk Foundation Center for Biosustainability, Technical University of Denmark, Kongens Lyngby, Denmark; e Genome Center, University of California, Davis, Davis, California, USA; NYU School of Medicine

**Keywords:** transcriptional regulatory network, adaptive evolution, metabolism, regulatory network, systems biology

## Abstract

Genotype-fitness maps of evolution have been well characterized for biological components, such as RNA and proteins, but remain less clear for systems-level properties, such as those of metabolic and transcriptional regulatory networks. Here, we take multi-omics measurements of 6 different E. coli strains throughout adaptive laboratory evolution (ALE) to maximal growth fitness. The results show the following: (i) convergence in most overall phenotypic measures across all strains, with the notable exception of divergence in NADPH production mechanisms; (ii) conserved transcriptomic adaptations, describing increased expression of growth promoting genes but decreased expression of stress response and structural components; (iii) four groups of regulatory trade-offs underlying the adjustment of transcriptome composition; and (iv) correlates that link causal mutations to systems-level adaptations, including mutation-pathway flux correlates and mutation-transcriptome composition correlates. We thus show that fitness landscapes for ALE can be described with two layers of causation: one based on system-level properties (continuous variables) and the other based on mutations (discrete variables).

**IMPORTANCE** Understanding the mechanisms of microbial adaptation will help combat the evolution of drug-resistant microbes and enable predictive genome design. Although experimental evolution allows us to identify the causal mutations underlying microbial adaptation, it remains unclear how causal mutations enable increased fitness and is often explained in terms of individual components (i.e., enzyme rate) as opposed to biological systems (i.e., pathways). Here, we find that causal mutations in E. coli are linked to systems-level changes in NADPH balance and expression of stress response genes. These systems-level adaptation patterns are conserved across diverse E. coli strains and thus identify cofactor balance and proteome reallocation as dominant constraints governing microbial adaptation.

## INTRODUCTION

Since 2006, adaptive evolution has been studied in controlled laboratory environments where whole-genome sequencing and fitness measurements are performed (1). The process of evolution can be studied on either long-term or short-term time scales. On long time scales, the focus is on understanding the dynamics of subpopulations (2 to 5). Adaptive laboratory evolution enables studying evolution on short time scales, which allows for the identification of few causal mutations that underlie increased fitness. Laboratory evolution of bacteria offers the possibility to achieve a multiscale description of evolutionary landscapes deduced through multi-omic measurements (6, 7).

Notably, many adaptive laboratory evolution (ALE) studies have performed genetic perturbations, such as gene deletions, in order to dissect how microbes adapt to altered conditions (7, 8). By taking 13-C fluxomics and RNA-seq measurements of genetic perturbation ALEs, researchers have been able to identify pathways and enriched subsystems that change the most throughout ALE. However, it remains unclear whether these principles apply to different strains of the same species and whether the evolutionary background of the strain (i.e., wild-type [WT] environment) plays a role. Thus, there is an impetus for performing comparative ALEs for different strains in order to elucidate conserved adaptation principles.

Furthermore, integration of RNA-seq is challenging in ALE studies, which is primarily due to the large number of gene variables (>4,000), which makes statistical associations difficult. Moreover, while researchers often perform subsystem enrichments of differentially expressed genes in order to gain insight into regulatory activity, such approaches lack quantitative details of regulatory activity that would enable other types of analysis such as identifying regulatory trade-offs and mutation correlates. Thus, the challenge to extract underlying principles from these high-dimensional data types remains, calling for more effective data-analysis methods. It has recently been shown that independent component analysis (ICA) leads to substantial dimensionality reduction in transcriptomics data sets through the identification of independently modulated sets of genes (called iModulons), opening the possibility to quantitatively interpret transcriptomic data sets. iModulons have provided a detailed understanding of changes in transcriptome composition in response to environmental and genetic perturbations (9 to 12). We thus sought to reveal multiscale adaptation principles in the E. coli species by taking multi-omics measurements of multiple E. coli strains throughout their adaptive laboratory evolution and capitalize on the systems-level insights that fluxomics data and iModulon analytics offer.

## RESULTS

### Consistent genetics in evolution of multiple E. coli strains.

**FIG 1:**
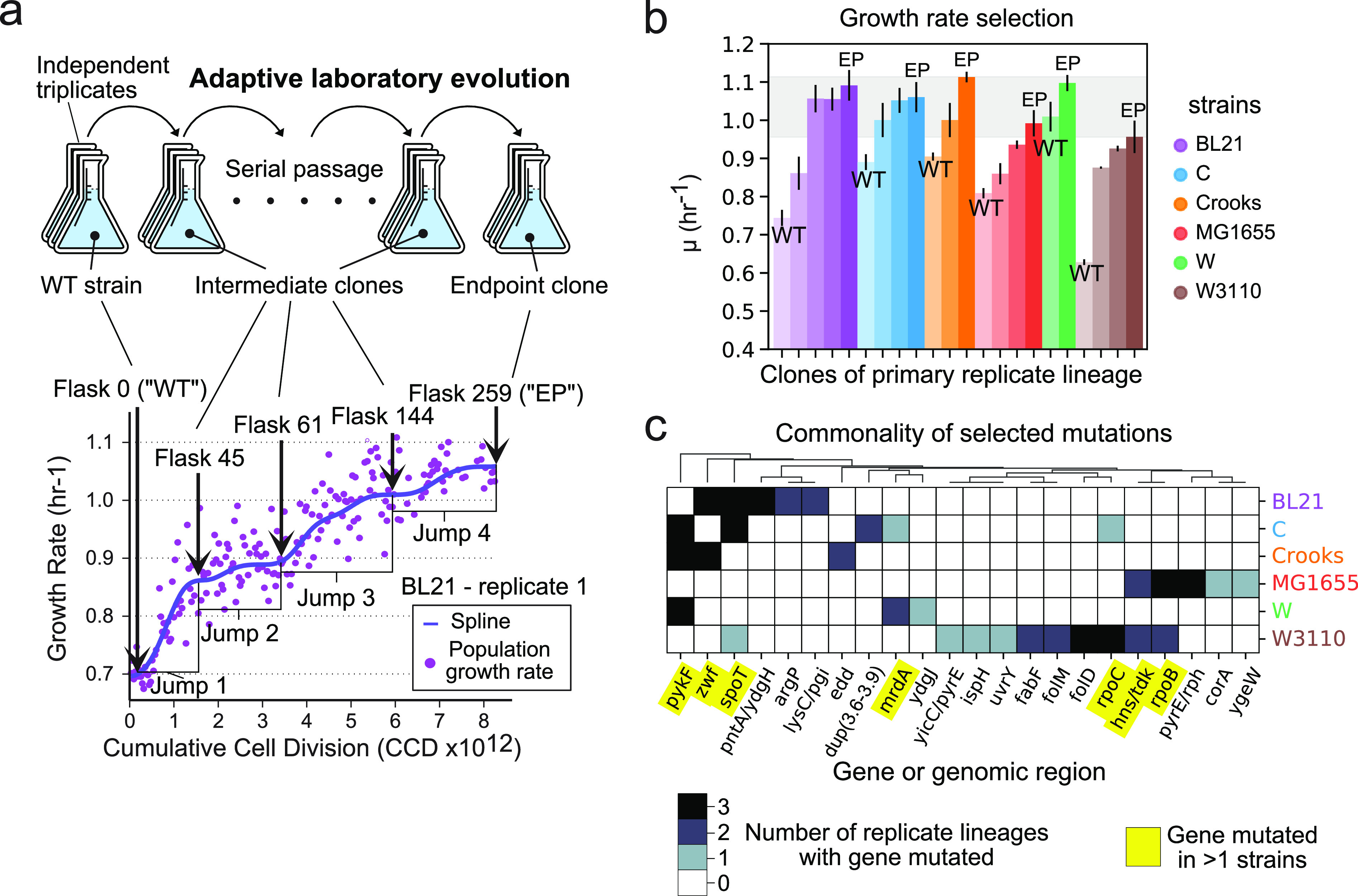
Overview of experimental design and key adaptation features. (A) Adaptive laboratory evolution was performed for each strain using independent triplicates. The wild-type (WT), evolved-intermediate, and evolved-endpoint (EP) clones underwent multi-omics measurements. An example growth curve is given for BL21 replicate 1 to illustrate WT, evolved-intermediate, and evolved EP flasks. (B) Bar plot of measured growth rates for wild-type, intermediate, and endpoint flasks for each strain. The error bars represent the standard deviation between the three triplicate flasks. (C) Heatmap of gene-level mutation frequency across replicate lineages of each strain. The intergenic region between two genes is noted by a slash (“/”).

Six different E. coli wild-type strains (K-12 MG1655, K-12 W3110, BL21, C, W, and Crooks) of different phylogroups were subjected to adaptive laboratory evolution (ALE) to select for rapid growth. We previously characterized the genetic and metabolic content of these strains and found significant genetic and metabolic variation (13). Triplicate independent lineages of each strain were evolved under a strict selection pressure for growth rate (Materials and Methods section and Fig. 1a; see Fig. S1 at https://figshare.com/articles/figure/Supplementary_Figure_1/20817613). Whole-genome sequencing was performed for populations and clones of all replicate lineages, while 13-C fluxomics, RNA-seq, and physiological measurements were performed for representative mutant clones from each starting strain at different growth rates (Fig. 1a).

We find that all strains start with different growth rates but evolve to rates ranging between 0.98 and 1.11 h^−1^ (D_t_ = 38 to 42 min) (Fig. 1b). Some strains (W and Crooks) operate near the maximal growth rates in their wild-type state, while others require multiple mutations to achieve the observed optimal growth rate (MG1655, W3110, BL21, and C).

We observed consistency in mutated genes along the growth trajectories, where each strain had at least one gene with a selected mutation in all replicate lineages (Fig. 1c). A total of seven genes (*pykF*, *zwf*, *spoT*, *mrdA*, *hns/tdk*, *rpoC*, *rpoB*) had selected mutations appear both in evolutions from multiple starting strains and in more than one replicate. Commonly mutated genes (Fig. 1c) were modified via SNPs only (*spoT*, *rpoC*, *rpoB*, *mrdA*), or additionally by indels (*pykF*), and mobile element insertions and larger deletions (*zwf*) (Data Set S1). The functionality of the mutated genes includes RNA polymerases (*rpoB*, *rpoC*), ppGpp synthetase (*spoT*), NADPH dehydrogenase (*pntA/ydgH*), pentose phosphate metabolism (*zwf*), glycolysis (*pykF*), folate metabolism (*folM*, *folD*), and cell wall (*mrdA*) (see Text S1 for further details of the selected mutations). The commonality of selected mutations indicated similar evolutionary constraints facing these strains and motivated an inquiry of their metabolic and gene expression profiles.

10.1128/msystems.00165-22.1 DATA SET S1 ALE mutation data for all evolutions. Download Data Set S1, CSV file, 0.02 MB. Copyright © 2022 Kavvas et al. 2022 Kavvas et al. https://creativecommons.org/licenses/by/4.0/ This content is distributed under the terms of the Creative Commons Attribution 4.0 International license.

10.1128/msystems.00165-22.5 TEXT S1 Notes describing details of the strain-specific ALE experiments. Download Text S1, DOCX file, 0.01 MB. Copyright © 2022 Kavvas et al. 2022 Kavvas et al. https://creativecommons.org/licenses/by/4.0/ This content is distributed under the terms of the Creative Commons Attribution 4.0 International license.

**FIG 2:**
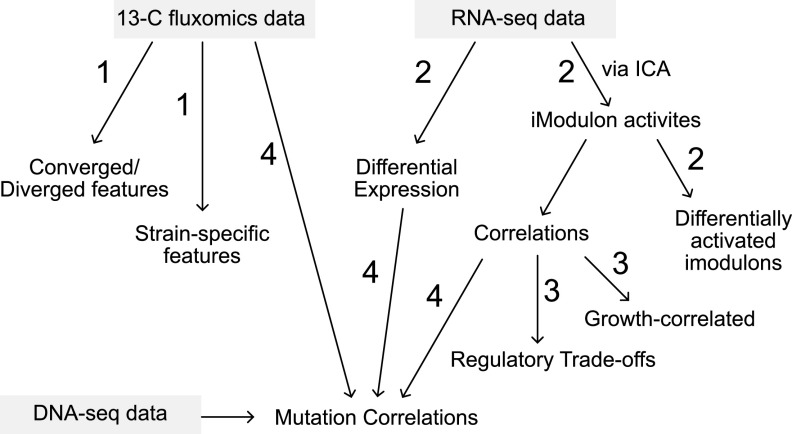
Overview of data analysis workflow. The figure portrays the steps of the analysis workflow where arrows describe the direction of analysis and the numbers describe the order at which they appear in this study. The numbers are grouped according to their general location in this study and appear in the same figure.

Our analysis from this point on consists of the following steps (Fig. 2): (i) an analysis of the fluxomics data, resulting in an understanding of converged and diverged metabolic features; (ii) an analysis of the RNA-seq data through differential expression and iModulon analysis, resulting in identification of genes and subsystems that changed most throughout the ALEs; (iii) a correlation analysis between iModulon activities, resulting in hypothesized regulatory trade-offs underlying adaptation; and (iv) a correlation analysis between ALE mutations and both fluxomic and RNA-seq data types, resulting in a link between causal mutations and system variables. We start with an analysis of the metabolic features.

### Characteristics of physiological and metabolic adaptations.

**FIG 3:**
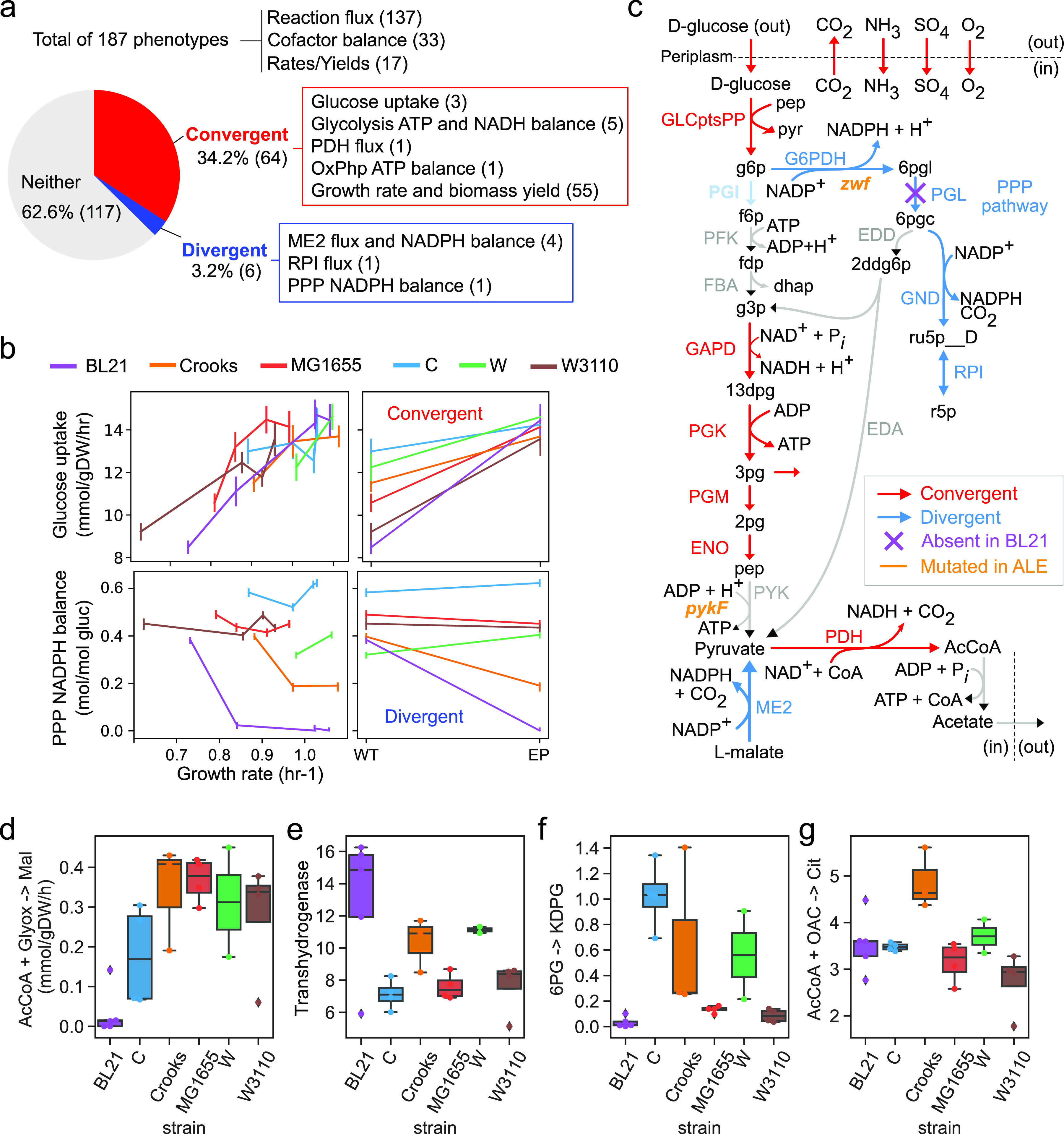
Adaptation in physiology and metabolism. (a) Pie chart describing the fraction of phenotypes that converge or diverge. Numbers in parentheses describe the number of related phenotypes. (b) Line plots of glucose uptake (top) and PPP NADPH balance (bottom) versus growth rate. Line plots and frequency distributions for WT and EP are plotted to the right for both cases. The error bars for glucose uptake describe standard deviation while the error bars for PPP NADPH balance describe 95% confidence intervals. (c) Metabolic map of reactions in glycolysis, PPP, and exchange reactions colored according to whether they diverge or converge. Blue describes divergence and red describes convergence. (d to g) Box plots of four reaction fluxes (absolute) that have strain-specific distributions. The dots represent outliers that are past 1.5 times the low and high quartiles. abs, absolute flux (mmol/gDW/hr); rel, relative flux (mol/mol glucose); TCA, citric acid cycle; PPP, pentose phosphate pathway; ME2, malic enzyme; OxPhp, Oxidative phosphorylation; PDH, pyruvate dehydrogenase.

Since a total of eight selected mutations were in genes encoding metabolic enzymes—two of which appear in multiple strains (*zwf*, *pykF*)—we hypothesized that, in spite of their different gene portfolios (13) (see Fig. S2 at https://figshare.com/articles/figure/Supplementary_Figure_2/20818270), the strains are evolving toward similar metabolic states. We thus set out to examine convergent and divergent phenotypes along the evolutionary trajectory by performing statistical tests for each physiological and fluxomic measurement between the wild-type (WT) and endpoint (EP) flasks for each strain (Materials and Methods). Of the 187 total phenotypes, 64 were identified as convergent across all strains (i.e., moved closer together during adaptation) and 6 were identified as divergent (i.e., moved further apart) with a false discovery rate (FDR) of less than 5% (Fig. 3a). Of the convergent phenotypes, we find that 86% (55/64) were growth-correlated (FDR < 0.05; Table S1).

10.1128/msystems.00165-22.6 TABLE S1 Statistics of convergent, divergent, and growth correlated phenotypes. Download Table S1, CSV file, 0.01 MB. Copyright © 2022 Kavvas et al. 2022 Kavvas et al. https://creativecommons.org/licenses/by/4.0/ This content is distributed under the terms of the Creative Commons Attribution 4.0 International license.

Convergent features were related to glucose uptake, glycolysis, and oxidative phosphorylation, while the top-ranked divergent features relate to NADPH production through differences in the use of the malic enzyme (ME2) and pentose phosphate pathway (PPP) (Fig. 3a). Examination of the ALE trajectories for the most convergent (Mann-Whitney U > 169, *P < *5.7 × 10^−5^) and divergent (Mann-Whitney U = 19, *P = *5.7 × 10^−5^) phenotypes showed that phenotypes do not monotonically increase/decrease during ALE (i.e., not always increasing or decreasing along the entire trajectory) (Fig. 3b). For example, although the glucose uptake rate has a significant net increase between WT and EP strains, four of the strains have one time period where glucose uptake decreases.

Principal-component analysis of metabolic fluxes showed that two components explain 93% of the variation and correspond to ATP production through oxidative phosphorylation and glycolysis (80%), and NADPH balance through pentose phosphate pathway and transhydrogenases (13%) (see Fig. S3 at https://figshare.com/articles/figure/Supplementary_Figure_3/20818351). These metabolic alterations are consistent with the ALE of E. coli metabolic knockout strains (8).

To determine whether specific metabolic reaction fluxes distinguish specific strains (WT or evolved), we tested all fluxes for strain-specific distributions and found four subsystems specific to BL21, Crooks, and C (ANOVA F-test, FDR < 0.05). The BL21 strain uniquely had no flux through the glyoxylate shunt while having the highest flux through transhydrogenase (Fig. 3d and e). Since the BL21 strain has low NADPH generation through PPP due to a lack of the pgl gene encoding 6-phoshpo-gluconolactonase (PGL) activity (14, 15), the high transhydrogenase flux likely compensates to regenerate NADPH. Furthermore, we find that all BL21 flask lineages select for mutations in the intergenic region of a transhydrogenase (*pntA/ydgH*) (Fig. 3d and e). We tested for correlations between *pnta/ydgh* mutations and alterations in gene expression, regulatory activities, and metabolic fluxes. We did not compute potential effects of *pnta/ydgh* mutations on functional features such as promoter motifs, binding sites, or ribosome binding sites (RBSs).

The C strain uniquely had high flux through the Entner-Doudoroff (ED) pathway, while BL21, MG1655, and W3110 had almost none (Fig. 3f). Crooks uniquely had the highest flux through tricarboxylic acid cycle (TCA) (Fig. 3g). In total, these results describe convergent and divergent metabolic traits that are either conserved or distinguish strains.

### Characteristics of gene expression adaptation.

**FIG 4:**
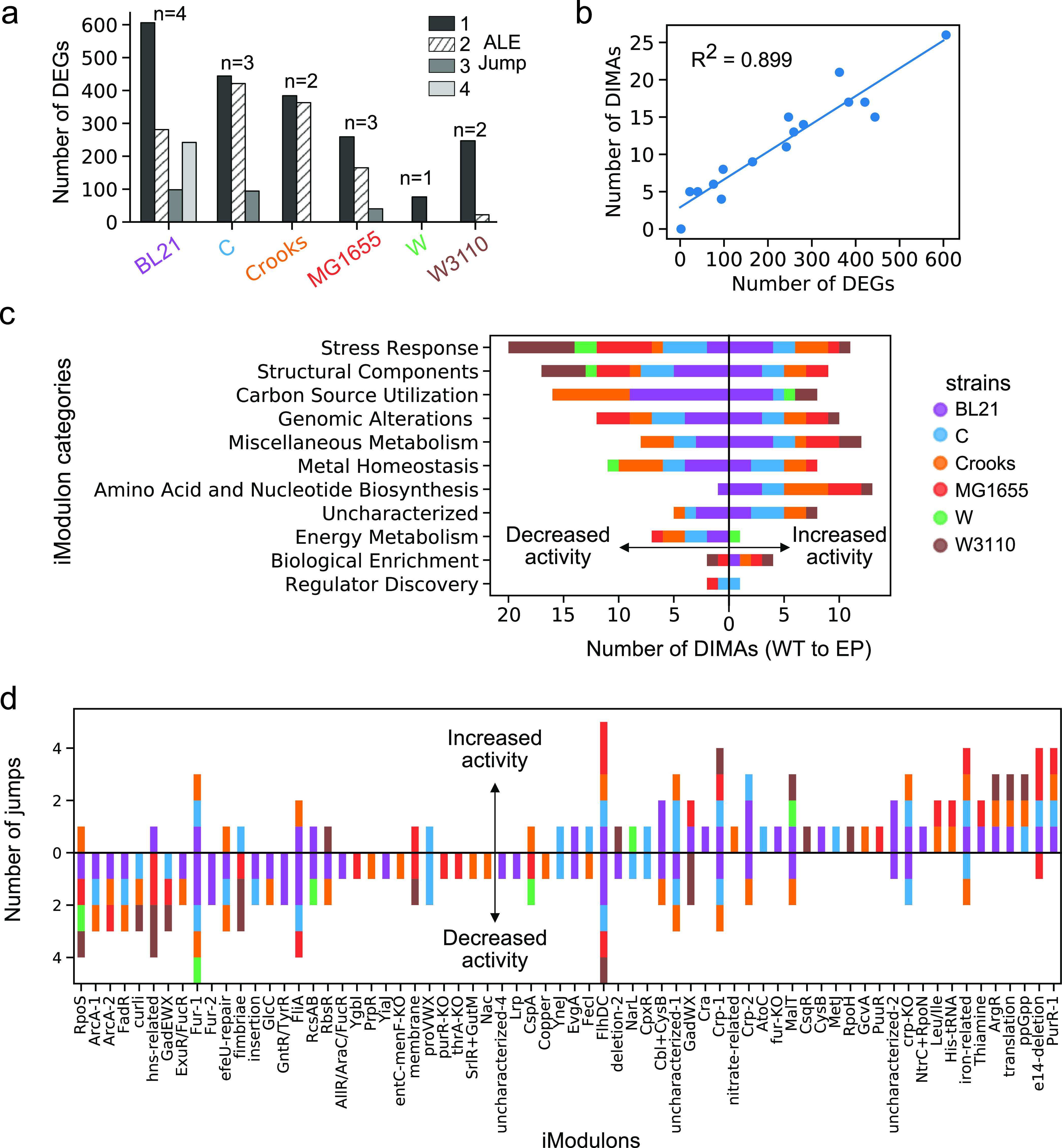
Characterization of gene expression adaptations. (a) Number of differentially expressed genes (DEGs) for each strain-specific jump in growth rate during ALE. (b) Scatterplot of the number of DEGs versus the number of differential iModulon activations (DIMAs) for all ALE jumps. (c) Bar plot of increased and decreased DIMA count in 11 functional categories. The count is summed across the six strains, ranked from top to bottom by the total number of times they were differentially activated in all ALEs. (d) Bar plot of DIMA counts for all iModulons separated into increased (top) and decreased (bottom) activities. The iModulons are ordered from left to right by the net sum of positive and decreased DIMAs (e.g., number increased to number decreased).

Associated with the metabolic differences between the six strains are differences in gene expression. We thus set out to analyze the transcriptomes of these strains by performing both differential expressed gene (DEG) and iModulon analyses for each lineage. The number of DEGs generally decreases along the adaptive evolution trajectory, with the exception of the last clone of BL21 isolated from the trajectory (Fig. 4a). iModulons, in contrast with DEGs that rely on thousands of variables, describe the composition of the transcriptome with just a few dozen variables (9). Specifically, iModulons simplify the dimensionality of the transcriptomics data set 20-fold in comparison to classical DEG analysis (Materials and Methods; Fig. 4b). iModulons describe sets of genes that are independently modulated across a compendium of transcriptomic data sets (9). The 92 iModulons, many associated with known transcription factors, have been described in the E. coli transcriptome and thus significantly simplify the challenge of interpreting transcriptomic changes during ALE (see Fig. S4 at https://figshare.com/articles/figure/Supplementary_Figure_4/20818399 for a general overview of iModulons).

In order to understand the different starting points of the six strains, we sought to identify iModulons that distinguish WT expression profiles for each strain independently of the others using an ANOVA F-test. We identified a total of 38 differentially activated iModulons (DIMAs) (Table S2, FDR < 0.005). For BL21, the iModulons suggest an original environment that was cold (cspA), anaerobic, and nitrate rich (ArcA-2), with gluconate (GntR/TyrR), allantoin, fructose, and arabinose (AllR/AraC/FucR) as possible carbon sources. For C, the identified iModulons point to a background with high acidity and osmotic stress (EvgA, proVWX). The low OxyR activity in Crooks implies a WT environment facing low oxidative stress, while high FliA activity in MG1655 suggests that high motility was advantageous to its original environment. The relatively high GadEWX in W3110 implies an original environment with high acid stress.

10.1128/msystems.00165-22.7 TABLE S2 iModulons distinguishing wild-type gene expression states for each strain. Download Table S2, CSV file, 0.00 MB. Copyright © 2022 Kavvas et al. 2022 Kavvas et al. https://creativecommons.org/licenses/by/4.0/ This content is distributed under the terms of the Creative Commons Attribution 4.0 International license.

iModulons thus give insights into the differential transcriptomic state of the wild types. We next sought iModulon insights into the adaptive evolutionary process itself. We performed differential iModulon activity analysis between the WT and EP flasks of each strain (Materials and Methods). We find a total of 57 iModulons that were differentially activated at least once among the different strains (*P* < 0.05, FC > 2) (i.e., significantly increase or decrease in activity). The most commonly activated iModulons corresponded to stress response and structural components (Fig. 4c). We find iModulons corresponding to amino acid and nucleotide biosynthesis to have mostly positive differentially activities. The W3110 strain had the largest number of differentially activated stress response iModulons, while BL21 had the most activated amino/nucleic acid biosynthesis iModulons. With respect to the total number of differentially activated iModulons, we find that BL21 has the most while W has the least, which reflects their respective change in growth rate. Of those activated, we find decreased activity in iModulons describing stress response (RpoS, GadEWX, RpoH, hns-related, proVWX) and motility (FlhDC, FliA, curli, fimbriae, RcsAB), with increased activity in iModulons describing translation machinery (translation) and amino acid and nucleic acid biosynthesis (PurR, ArgR, His-tRNA) (Fig. 4d).

### Conserved growth-dependent transcriptome adaptations.

iModulons that correlate with growth-rate dependence in all strains represent processes that drive increased growth rate in all strains. We found six such iModulons (Fig. 2a; median Pearson |R| > 0.75, median *P* value < 0.05). Of the six, three are positively correlated with growth rate and describe the expression of ribosomal genes (translation), arginine biosynthetic genes (ArgR), and nutrient response (ppGpp). The other three iModulons are negatively correlated with growth rate and describe stress response (RpoS, GadEWX) and structural assembly (curli). These iModulons describe growth-dependent transcriptome adaptations that are strongly conserved in the E. coli species, in spite of the significant differences between the strains’ genomes.

### Conserved transcriptomic trade-offs governing adaptation.

The identification of both positively and negatively growth-correlated iModulons implies the existence of trade-offs; i.e., increased expression of certain genes requires decreased expression of others. To identify the dominant tradeoffs, we filtered for iModulon test pairs by performing principal-component analysis (PCA) of the iModulon activities that changed during “jumps” in fitness during ALE (Materials and Methods). A jump is defined as the difference in growth rate between two assayed clones with a defined genotype.

**FIG 5:**
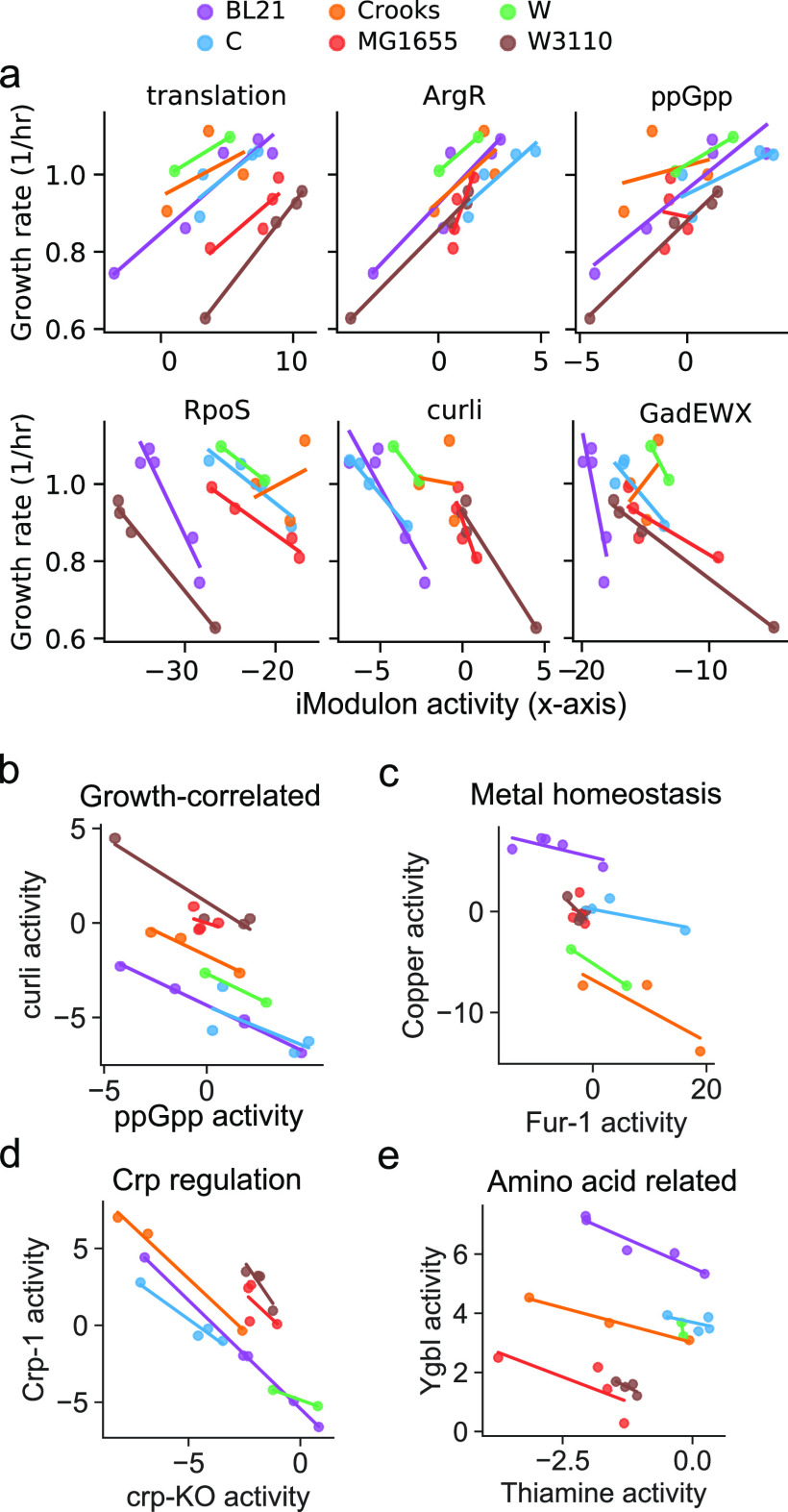
Conserved transcriptomic adaptations in the six strains studied. (a) Strain-specific line plots of growth rate versus iModulon activity for six iModulons (median Pearson |R| > 0.75, median *P* value < 0.05). (b to e) Strain-specific line plots for iModulon activities for trade-offs reflecting growth-correlated iModulons, metal homeostasis, Crp regulation, and amino acid-correlated iModulons (ANCOVA R^2^ > 0.95, FDR < 0.05).

**FIG 6 fig6:**
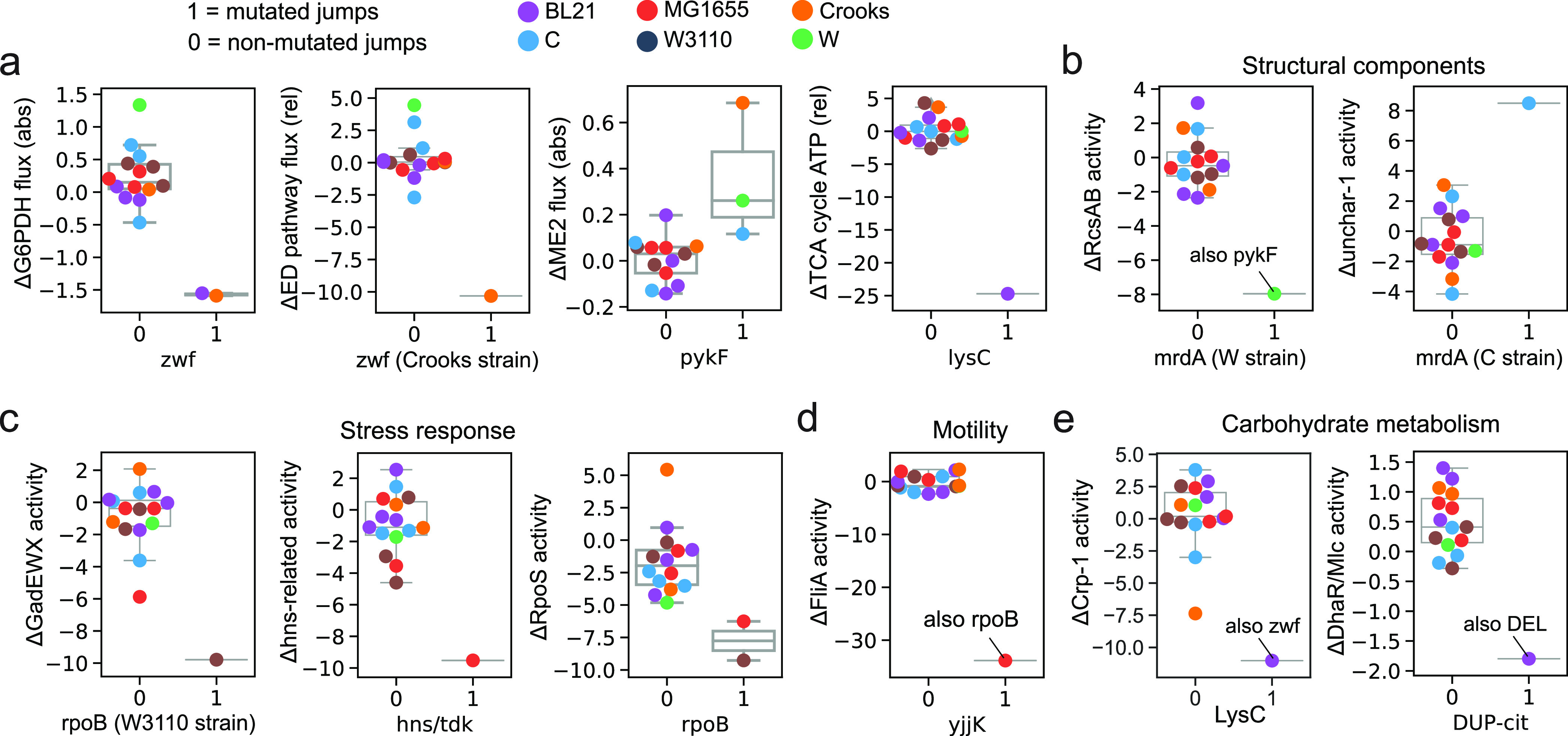
Mutation correlates. (a) Boxplots of significant correlations between mutations and changes in metabolic fluxes. The terms “abs” and “rel” in parentheses refer to absolute flux (mmol/gDw/h) and relative flux (mol/mol gluc), respectively. (b to e) Boxplots of significant correlations between mutations and changes in iModulon activities. The boxplots are grouped by iModulon functional category. Genes with strains in parentheses note a strain-specific mutation correlation. Mutations are grouped at the gene level unless noted otherwise.

We find that the first two PCA components explain the majority of the variance and have an explained variance ratio of 43% and 19%, respectively (see Fig. S5 at https://figshare.com/articles/figure/Supplementary_Figure_5/20818438). The first component describes metal-related iModulons (Fur-1, Fur-2, iron-related, efeU-repair, Copper) and growth-correlated iModulons (RpoS, translation, ppGpp). The second component primarily describes carbon-metabolism iModulons (Crp-1, Crp-2, MalT) with positive weight and stress response and structural iModulons with opposite weight (RpoS, GadWX, GadEWX, hns-related, CspA, curli). Using the PCA-filtered list of iModulons, we tested for negative correlations and identified 11 negatively correlated iModulon pairs (ANCOVA R^2^ > 0.95, FDR < 0.05) that fall into four trade-off groups: growth-correlated (curli versus translation, ppGpp, ArgR), metal homeostasis (Copper versus Fur-1), Crp regulation (Crp-KO versus Crp-1, Crp-2), and amino acid biosynthesis (YgbI versus Thiamine, His-tRNA, Leu/Ile, purR-1) (Fig. 5b [Fig fig6]to e).

These results reveal clear strategies in the adjustment of the transcriptome composition that in turn reflect the changes in proteome allocation required to achieve optimal fitness. They also reveal systems-level mechanisms underlying the fitness landscape.

### Causal mutation correlates elucidate systems-level adaptations.

Conversely, at the genetic level, comparing mutations is challenging due to the significant genomic differences between strains. We therefore leveraged fitness jumps by looking at differences between flasks (i.e., selected mutations with changes in flux and iModulon activities for a specific jump) instead of flasks themselves (i.e., all mutations in a flask with flux and iModulon activities for the flask). This allows us to focus on comparing causal mutations with their relative effects on the systems level. Using these jump-specific changes, we could search for associations between jump-specific differences in both reaction flux and iModulon activity, with the coincident selection of mutations at both the nucleotide and gene levels.

We find four flux correlations primarily describing reactions involved in cofactor balancing (FDR < 5%) (Fig. 6a). Specifically, *zwf* mutations are correlated with ΔG6PDH flux (NADPH balance through the PP pathway), *pykF* mutations with ΔME2 flux (NADPH balance through malic enzyme), and *lysC* with ΔSUCCOAS flux (ATP and NADPH balance through the TCA cycle). We find that the *zwf* mutation in Crooks is uniquely associated with ΔED pathway flux.

We identify eight mutation correlates that fall into four different iModulon functional categories describing stress response, motility, structural components, and carbohydrate metabolism (FDR < 5%) (Fig. 6b to e). Notably, no correlations could be derived between mutations and DEGs, which exemplifies the efficacy of iModulon-based transcriptome analysis.

## DISCUSSION

In summary, we provide a mechanistic understanding of fitness landscapes by revealing the metabolic and transcriptomic principles underlying adaptations of E. coli strains. On the metabolic level, the strains similarly adapted their ATP and NADH production strategies, but differed in their NADPH production strategy. This divergence had a genetic basis, with significant correlations identified between changes in NADPH-producing reaction fluxes and *zwf* and *pykF* mutants. The evolutionary plasticity of NADPH balancing may be attributed to the inherent flexibility in the structure of the metabolic network, where it has been shown that NADPH production can be easily switched between pathways (16). On the transcriptomic level, the strains similarly increased ribosomal and amino acid biosynthetic genes and decreased expression of stress response and structural components. The altered regulatory systems capture a large portion of the transcriptome, and thus likely represent adaptation toward efficient proteome utilization (17). We found 7 mutations to be correlated with these conserved transcriptome reallocations. These mutations delineate based on strain phylogeny and thus may reflect the evolutionary background of the wild-type strains used that predetermined their transcriptional regulation. Notably, the identification of NADPH and stress response came out of an entirely data-driven analysis of the RNA-seq and MFA data set. While the RNA-seq data set composed a large percentage of the total transcriptome, the metabolic flux data were limited to central pathways. The majority of metabolic mutations appeared in these pathways, which suggests that our MFA data encapsulate the key evolutionary constraints. Our analysis focused on the regulation of gene expression but left out regulation at the level of translation and posttranslational modifications. Future studies may perform *in situ* detection methods or Western blots toward elucidating adaptation at these regulatory levels. The overall results point to cofactor balance and transcriptome allocation as the dominant constraints governing E. coli adaptation and reveal the strategies underlying their adjustment to develop increased fitness.

**FIG 7:**
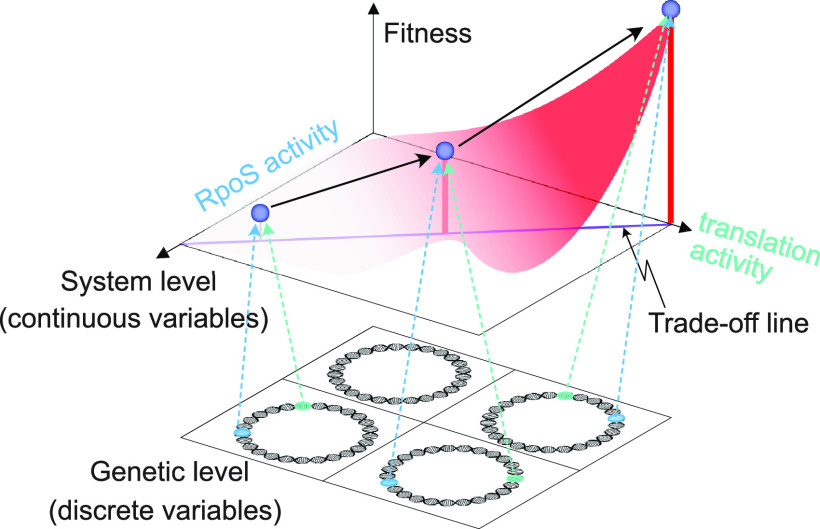
Two-level description of bacterial fitness landscape. Our results identify causal mutations that underlie specific changes in the gene regulatory network (see Fig. 3). We find that trade-offs govern alterations in the gene regulatory network for all strains, where increased activity of an iModulon comes at the cost of decreased activity of another (see Fig. 2). The circles represent different genomes.

Broadly speaking, this study suggests how two levels of underlying mechanisms can be developed to interpret the fitness landscape for bacterial adaptation. One is in terms of systems parameters and the other in terms of genetic parameters (Fig. 7). At the systems level, we have defined continuous variables, the iModulon activities, that represent adaptations in the composition of the transcriptome. Conversely, at the genetic level, the mutations are discrete as they represent well-defined sequence changes. In a broad sense, this two-level decomposition parallels the one used in physics to distinguish between the continuum- and molecular-level descriptions of physical phenomena.

## MATERIALS AND METHODS

### Adaptive laboratory evolution and DNA sequencing.

ALE was performed using 3 independent replicates of each strain. Cultures were serially propagated on M9 minimal medium (18) with 2 g/L glucose at 37°C and well mixed for proper aeration using an automated system (18, 19) that periodically passed 150 μL of the cultures to a new fresh 30-mL flask with a total working volume of 15 mL M9 medium (i.e., a 1:100 ratio) once they had reached an optical density (OD_600_) of 0.3 (Tecan Sunrise plate reader, equivalent to an OD_600_ of 1.3 on a traditional spectrophotometer with a 1-cm path length). Such a routine to pass at the late exponential phase of growth, was to keep cells under constant selection pressure for higher fitness, i.e., growth rate. Cultures were always maintained in excess nutrient conditions assessed by nontapering exponential growth. The laboratory evolution was performed for a sufficient time interval to allow the cells to reach its fitness plateau. Periodically, glycerol cryogenic stocks were prepared and stored at −80°C for any culture restarting. The fitness jump was observed in about 200 generations; however, the experiment was continued for approximately 900 generations to explore the possibility of any secondary fitness jump. Further passaging was stopped due to the absence of any appreciable growth rate increase in about 700 generations. The slope of ln(OD_600_) versus time of four OD_600_ measurements from each flask was used to determine the growth rate. A cubic interpolating spline constrained to be monotonically increasing was fit to these growth rates to obtain the smoothed fitness trajectory curves. DNA resequencing was performed on a clone from the end points of evolved strains as described earlier by Lacroix et al., 2015 (19). The ALE mutation data are provided for all replicate lineages (Data Set S1).

### Flask terminology and selection.

The term *flask* is a count that describes evolutionary time. The evolutions were initiated with a single colony in “Flask 0,” i.e., a preculture, and then each successive flask grows by 1 until the end of the evolution experiments.

A clone was chosen out of a population at a given flask number and was compared to population sequencing from the identical flask. Given that three independent evolutions for each starting strain were performed, and that there was a high degree of parallel evolution (see Data Set S7), it was possible to choose a given clone that well represented the dominant genotype of any given starting strain at the chosen evolutionary time points, i.e., flasks. Thus, to answer the critique directly, interclonal differences in a given evolved population were excluded out of a desire to simplify the study.

RNAseq was performed on a given isolated clone where a pure culture starting from a single colony was grown to an appreciable OD that was sufficient to perform the assay. It was assumed that there were no major mutations selected for during such culturing over a few generations to collect the requisite amount of biomass.

### RNA sequencing and processing.

Total RNA was sampled from duplicate cultures. Growth curve analysis was performed using a Bioscreen C Reader system with 20 μL culture volume per well. Two biological replicates were used in the assay. Media components were purchased from Sigma-Aldrich (St. Louis, MO). After inoculation and growth, 3 mL of cell broth (OD_600_ ~0.5) was immediately added to two volumes of Qiagen RNA-Protect Bacteria Reagent (6 mL), vortexed for 5 s, incubated at room temperature for 5 min, and immediately centrifuged for 10 min at 11,000 × *g*. The supernatant was decanted, and the cell pellet was stored in the −80°C. Cell pellets were thawed and incubated with ReadyLyse Lysozyme, SuperaseIn, Protease K, and 20% SDS for 20 min at 37°C. Total RNA was isolated and purified using the Qiagen RNeasy minikit columns and following vendor procedures. An on-column DNase treatment was performed for 30 min at room temperature. RNA was quantified using a Nanodrop and quality assessed by running an RNA-nano chip on a bioanalyzer. The rRNA was removed using Illumina Ribo-Zero rRNA removal kit for Gram-negative bacteria. A KAPA stranded RNA-Seq kit (Kapa Biosystems KK8401) was used following the manufacturer’s protocol to create sequencing libraries with an average insert length of around ~300 bp. Libraries were run on a HiSeq4000 (Illumina). All RNA-seq experiments were performed in biological duplicates from distinct samples. Raw-sequencing reads were deposited to GEO (see Data Set S2 for accession numbers).

10.1128/msystems.00165-22.2 DATA SET S2 Genome reference IDs, GEO accession numbers, and metadata. Download Data Set S2, CSV file, 0.01 MB. Copyright © 2022 Kavvas et al. 2022 Kavvas et al. https://creativecommons.org/licenses/by/4.0/ This content is distributed under the terms of the Creative Commons Attribution 4.0 International license.

Raw-sequencing reads were mapped to the reference genomes (see Data Set S2 for reference genome numbers) using Bowtie (v1.1.2) (20) with the following options: “-X 1000 -n 2 −3 3.” Transcript abundance was quantified using summarizeOverlaps from the R GenomicAlignments package (v1.18.0), with the following options: “mode = “IntersectionStrict,” singleEnd = FALSE, ignore.strand = FALSE, preprocess.reads = invertStrand” (21). To ensure the quality of the compendium, genes shorter than 100 nucleotides and genes with under 10 fragments per million-mapped reads across all samples were removed before further analysis. Transcripts per million (TPM) were calculated by DESeq2 (v1.22.1) (22). The final expression compendium was log-transformed log_2_(TPM + 1) before analysis, referred to as log-TPM (Data Set S3). Biological replicates with R2 of <0.9 between log-TPM were removed to reduce technical noise.

10.1128/msystems.00165-22.3 DATA SET S3 Gene expression data of the core genome for each strain in log(TPM). Download Data Set S3, CSV file, 1.7 MB. Copyright © 2022 Kavvas et al. 2022 Kavvas et al. https://creativecommons.org/licenses/by/4.0/ This content is distributed under the terms of the Creative Commons Attribution 4.0 International license.

### Fluxomics.

Metabolic characterization by 13C metabolic flux analysis was performed as described in references 23 and 24. Briefly, for ^13^C-tracer experiments, strains were cultured aerobically in glucose M9 minimal medium at 37°C in minibioreactors with 10 mL working volume. Precultures were grown overnight and then used to inoculate the experimental culture at an OD_600_ of 0.01, in which 2 g/L of [1,6-^13^C]glucose was present. Cells were harvested for gas chromatography mass spectrometry (GC-MS) analysis at midexponential growth when OD_600_ was approximately 0.7. [1,6-^13^C]glucose was previously identified as an optimal tracer for global flux resolution (25).

Chemicals and M9 minimal medium were purchased from Sigma-Aldrich (St. Louis, MO). Isotopic tracers were purchased from Cambridge Isotope Laboratories (Tewksbury, MA): [1,6–13C]glucose (99.2% isotopic purity, 99.7% chemical purity). The isotopic purity and enrichment of all tracers were validated by GC-MS analysis. All solutions were sterilized by filtration.

Samples were collected during the exponential growth phase to monitor cell growth, glucose consumption, and acetate production. Cell growth was monitored by measuring the optical density at 600 nm (OD_600_) using a spectrophotometer (Eppendorf BioPhotometer). The OD_600_ values were converted to cell dry weight concentrations using a predetermined OD_600_ dry cell weight relationship for E. coli (1.0 OD_600_ = 0.32 gDW/L) (26). After centrifugation, the supernatant was separated from the biomass pellet and glucose concentration was measured with a YSI 2700 biochemistry analyzer (YSI, Yellow Springs, OH). Acetate was measured by HPLC.

GC-MS analysis was performed on an Agilent 7890B GC system equipped with a DB-5MS capillary column (30 m, 0.25 mm internal diameter, 0.25 μm-phase thickness; Agilent J&W Scientific), connected to an Agilent 5977A mass spectrometer operating under ionization by electron impact (EI) at 70 eV. Helium flow was maintained at 1 mL/min. The source temperature was maintained at 230°C, the MS quad temperature at 150°C, the interface temperature at 280°C, and the inlet temperature at 250°C. GC-MS analysis of tert-butyldimethylsilyl (TBDMS) derivatized proteinogenic amino acids was performed as described (23). Labeling of glucose (derived from glycogen) and ribose (from RNA) were determined as described. In all cases, mass isotopomer distributions were obtained by integration (27) and corrected for natural isotope abundances (28). All mass isotopomer data are provided (Data Set S4).

10.1128/msystems.00165-22.4 DATA SET S4 Fluxomic measurements for all metabolic reactions, metabolite GC-MS, and data for the 13C-MFA. Download Data Set S4, CSV file, 0.1 MB. Copyright © 2022 Kavvas et al. 2022 Kavvas et al. https://creativecommons.org/licenses/by/4.0/ This content is distributed under the terms of the Creative Commons Attribution 4.0 International license.

The metabolic network model used for ^13^C-MFA is provided (Data Set S4). The model (24) includes all major metabolic pathways of central carbon metabolism, lumped amino acid biosynthesis reactions, and a lumped biomass formation reaction. ^13^C-MFA calculations were performed using the Metran software (29), which is based on the elementary metabolite units (EMU) framework (30). Fluxes were estimated by minimizing the variance-weighted sum of squared residuals (SSR) between the measured and model-predicted mass isotopomer distributions and acetate yield using nonlinear least-squares regression. Flux estimation was repeated 10 times starting with random initial values for all fluxes to find a global solution. At convergence, accurate 95% confidence intervals were computed for all estimated fluxes by evaluating the sensitivity of the minimized SSR to flux variations. Precision of estimated fluxes was determined as follows:

Flux precision (stdev) = [(flux upper bound 95%) − (flux lower bound 95%)]/4

To describe fractional labeling of biomass amino acids, G-value parameters were included in ^13^C-MFA. As described previously (23), the G-value represents the fraction of a metabolite pool that is produced during the labeling experiment, while 1-G represents the fraction that is naturally labeled (e.g., from inoculum). By default, one G-value parameter was included for each measured amino acid in each data set. Reversible reactions were modeled as separate forward and backward fluxes. Net and exchange fluxes were determined as follows: vnet = vf – vb; vexch = min(vf, vb). To determine the goodness of fit, 13C-MFA fitting results were subjected to a 2-statistical test (31).

### Mann-Whitney U tests for identifying convergent and divergent phenotypes.

To perform statistical tests for convergent and divergent features, we transformed the data vectors describing the mean physiological and fluxomics values for the size WT and EP flasks to vectors containing the pairwise distances among the points. The conversion resulted in a total of 15 points for each of the WT and EP flasks. The transformation to pairwise distances accounts for how close the strains were at each point (i.e., convergence describes points coming closer together). Mann-Whitney U tests were then carried out to test whether the EP pairwise distances are smaller than the WT pairwise distances (i.e., the EP values are closer together than the WT values). We calculated the *P* values using both a normal approximation and one implemented with the mannwhitneyu function in scipy stats. Both of the statistic estimates captured the general behavior, but the normal approximation was utilized due to the lack of table *P* values for U statistics less than 36. We then selected the convergent and divergent features as those with a false discovery rate (FDR) less than 5% using the Benjamini-Hochberg correction, implemented in the statsmodels package version 0.9.0 (32).

### Differential expression analysis of RNA-seq.

We performed differential expression analysis of the RNA-seq profiles between consecutive ALE flasks (i.e., ALE evolution stages) using the R package DESeq2 (22). Specifically, differential expression was performed for each pair of flasks describing the before and after of an ALE experiment. We utilized an adaptive t prior shrinkage estimator (33) to transform the log fold changes for better ranking and visualization of the differential expression results. We performed a sensitivity analysis of the *P* value and Log_2_ fold change thresholds on determining sets of significantly expressed genes.

### iModulon analysis of RNA-seq data.

We previously showed that independent component analysis (ICA) deconvolved a large compendium of E. coli MG1655 RNA-seq data into a linear combination of independent sources (“iModulons”) that reflect known regulons, and source weightings (“iModulon activities”), which describe the global regulatory state (9). The resulting matrix decomposition by ICA (9) is formulated as follows:

$${\text{X}}_{\text{PRECISE}}={\text{ M}}_{\text{PRECISE}}\cdot {\text{A}}_{\text{PRECISE}},$$

where X_PRECISE_ is the previously utilized PRECSE RNA-seq data described in transcripts per million (TPM), M_PRECISE_ is the matrix describing the iModulon gene sets (genes as rows and iModulons as columns), and A_PRECISE_ is the sample-specific iModulon activities (iModulons as rows and samples as columns). Using the previous set of 92 iModulons (M_PRECISE_), we transformed the flask-specific gene expression profiles of our six E. coli strain ALEs (X_6strain_) into flask-specific iModulon activities (A_PRECISE_) (see Fig. S3a at https://figshare.com/articles/figure/Supplementary_Figure_3/20818351), formulated as follows:

$${\text{A}}_{\text{6strain}}={\text{ M}}_{\text{PRECISE}}{}^{-\text{1}}\cdot {\text{X}}_{\text{6strain}},$$

where A_6strain_ and X_6strain_ describe the flask-specific iModulon activities and flask-specific gene expression TPM profiles, respectively (Table S3). The previously uncharacterized iModulons Uncharacterized-6, Uncharacterized-5, and Uncharacterized-3 were characterized in this study as hns-related, ppGpp, and CspA, respectively. Together, the 92 iModulons explained 52% of the expression variance of the multistrain core genome, where they explained the most expression for MG1655 (67.78%) and the least for C (44.23%) (see Fig. S5b at https://figshare.com/articles/figure/Supplementary_Figure_5/20818438). A figure illustrating the translation iModulon gene set is provided (see Fig. S5c at https://figshare.com/articles/figure/Supplementary_Figure_5/20818438).

10.1128/msystems.00165-22.8 TABLE S3 iModulon gene weights per sample in our dataset. Download Table S3, CSV file, 0.05 MB. Copyright © 2022 Kavvas et al. 2022 Kavvas et al. https://creativecommons.org/licenses/by/4.0/ This content is distributed under the terms of the Creative Commons Attribution 4.0 International license.

### Differential activity analysis of iModulons.

Distribution of differences in iModulon activities between biological replicates was first calculated, and a log-norm distribution was fit to the differences (10). In order to test statistical significance, absolute value of difference in activity level of each iModulon between the two samples was calculated. This difference in activity was compared to the log-normal distribution from above to get a *P* value. Because differences and *P* value for all iModulons were calculated, the *P* value was further adjusted with Benjamini-Hochberg correction to account for multiple hypothesis testing problems. Only iModulons with change in activity levels greater than 5 were considered significant. Differential activity analysis was performed for all ALE jumps as well as between the WT and EP flask for each strain.

### Data transformation to jump-specific perspective.

A jump-specific perspective of the data was taken for our iModulon PCA and mutation correlation analysis. Specifically, we transformed the activity matrix (flask specific) to the difference in flask activity along the trajectory (jump specific) in order to identify components describing general adaptation trends as opposed to strain differences. We formulate this as follows:

$$\Delta {\text{X}}_{\text{jump}i,\text{ strain}j}={\text{ X}}_{\text{flask i}+\text{1},\text{ strain}j}\;-\;X_{\text{flask}i,\text{ strain}j},$$

where ΔX describes the jump-specific data set with 16 rows (jumps) and X describes the original flask-specific data set with 22 rows (flasks).

### Trade-off analysis through PCA and ANCOVA.

In order to avoid harsh statistical corrections when testing all possible iModulon pairs, we performed PCA using the jump-specific iModulon activities in order to filter out a candidate set of iModulons for downstream correlation tests. Since our initial run of PCA resulted in the first component (explaining 40% of the variation) describing large FlhDC and FliA activity unique to the first MG1655 jump, we filtered out the FlhDC and FliA iModulon outliers. We then performed both analysis of covariance (ANCOVA) and Pearson correlation tests for iModulons that had PCA weights greater than 0.10 in components explaining at least 5% of the variation. ANCOVA was performed to test the similarity of the strain-specific regression lines (dependence on strain-specific categorization). Trade-offs were identified as iModulon pairs with ANCOVA R^2^ of >0.90.

### Code availability.

Code is available upon request.

### Data availability.

The physiological, fluxomics, and genome sequencing data sets generated and analyzed during this study are included in this published article (and its supplemental information files). The RNA-sequencing data sets generated during and analyzed during the current study are available in the SRA repository (https://www.ncbi.nlm.nih.gov/bioproject/PRJNA644668).
