# Supplementary material for: Experimental Evolution Reveals Unifying Systems-Level Adaptations but Diversity in Driving Genotypes
Source: mSystems. 2022 Oct 13;7(6):e00165-22. doi: 10.1128/msystems.00165-22 (PMC9765567; doi:10.1128/msystems.00165-22)
Supplement: TEXT S1 [file msystems.00165-22-s0008.docx]

***Supplementary Information* for Laboratory evolution reveals unifying systems-level adaptation mechanisms but diversity in driving genotypes**

Erol S. Kavvas^5^, Christopher P. Long^2†^, Anand Sastry^1^, Saugat Poudel^1^, Maciek R. Antoniewicz^2‡^, Yang Ding^1^, Elsayed T. Mohamed^4^, Richard Szubin^1^, Jonathan M. Monk^1^, Adam M. Feist^1,4,^**^*^**, Bernhard O. Palsson^1,3,4^**^*^**

## Supplementary Notes

**W ALE mutation notes**

- *pykF* mutated in each replicate and all had distinct mutations, one likely a loss of function given a out of frame 1 bp deletion
- In all replicates, there were mutations in genes associated with the peptidoglycan pathway (two distinct *mrdA* mutations and one in *mraY*).
- For one of replicates (replicate 2), there were no mutations which were fixing in the population, even after 75 flasks, likely due to the initial high growth rate.
- No mobile element insertion related mutations were detected, although IS elements are present in the genome.

**Crooks ALE mutation notes**

- All of the 3 replicates had mutations in *pykF* and *zwf*
- *zwf* mutations always appeared earlier in the evolution in the intermediate sequenced samples
- One of the populations was a hypermutator, likely due to a *mutH* mutation, but the same *zwf* and *pykF* mutational signals were seen in this clone.
- The same mutation in *edd* (A→G, L6L) was observed at lower frequencies in 2 replicates when examining population sequencing
- Marginal Mutation prediction: an IS element insertion in *pykF* in likely in A15_F118_I1 given high % in new junction evidence

**C ALE mutation notes**

- *spoT* was mutated after the first jump in fitness for all of the replicates. The same *spoT* mutation fixed in the final populations of 2/3 replicates, but there were 7 different SNPs in *spoT* detected across all of the replicates.
- The *pykF* gene was mutated after the second jump in fitness for all of the populations, 2 SNPs were detected and one 1 bp deletion.
- All of the replicates had a CNV in the first intermediate sample only that was not seen later as the evolution progressed. There was an overlap in the amplified region.
- No mobile element insertion related mutations were detected, although IS elements are present in the genome.

**BL21 ALE mutation notes**

- All of the evolutions mutated the *zwf* gene, early and often, some with likely loss of function mutations. Interesting, as its known that BL21 has lost its *pgl* gene that is directly downstream of *zwf* in the PPP. All of the samples resequenced had a *zwf* mutation.
- In conjunction with the *zwf* mutations, there were multiple hits affecting the *pgi* gene and the transhydrogenase (*pntA*). The *pntA* mutations appear after the *zwf* mutations, which implies this mutation is a secondary response of a *zwf* mutation, modulating the NADPH pool.
- *spoT* is mutated in all of the replicates, it appeared late in the evolutions in two out of three replicates.
- The *argP* gene also mutated in two of the replicates early (one at a frequency of 0.11 of the first replicate), but did not persist.

**W3110 ALE mutation notes**

- All of the populations had either an *rpoB* or *rpoC* mutation in every resequencing step. The same *rpoC* mutation (T757K) shows up in the initial first or second flasks, an *rpoB* mutation (H526Y) also shows up in 2 of the 3 replicates. This likely indicates they occurred in the preculture. Interestingly, none of the same *rpoB* or *rpoC* mutants persisted and were fixed in the final population.
- All of the replicates had the same 4bp deletion in the *folM* gene appear at some fraction, then be lost sometimes to another *folM* mutation or *folD* mutation. *folM* and *folD* catalyze reactions two steps away from each other in the folate biosynthesis pathway. The starting strain has a mutation in folD as compared to the reference sequence in NCBI.
- *spoT* shows up in one of the replicates population sequencing at a low frequency.
- *fabF* shows up in 2 out of 3 replicates
- Marginal Mutation prediction: *hns/tdk* mutations were detected, but had to be manually annotated in the replicates. The third replicate only appears to have one sample with an IS element mutation in this genomic region.

|  |  | | | |
| --- | --- | --- | --- | --- |
|  |  | | | |
|  |  | | | |
|  |  |  |  |  |
|  |  |  |  |  |
|  |  | | | |
|  |  | | | |
